# Transarterial chemoembolization with or without multikinase inhibitors for patients with unresectable hepatocellular carcinoma: a systematic review and meta-analysis of randomized controlled trials

**DOI:** 10.3389/fonc.2023.1139025

**Published:** 2023-06-08

**Authors:** Han Dong, Dongfang Ge, Biao Qu, Ping Zhu, Qibiao Wu, Tianyun Wang, Jue Wang, Zheng Li

**Affiliations:** ^1^ Department of Nursing, Huaian Hospital of Huaian City, Huaian, China; ^2^ President’s Office of Huaian Hospital of Huaian City, Huaian, China; ^3^ Department of Clinical Pharmacology, The Second Hospital of Anhui Medical University, Hefei, China; ^4^ Department of Endocrinology, Huaian Hospital of Huaian City, Huaian, China; ^5^ State Key Laboratory of Quality Research in Chinese Medicines, Faculty of Chinese Medicine, Macau University of Science and Technology, Macau, Macau SAR, China; ^6^ Department of Pharmacy, Huaian Hospital of Huaian City, Huaian, China; ^7^ College of Health Sciences, School of Life Sciences, Jiangsu Normal University, Xuzhou, China; ^8^ State Key Laboratory of Natural and Biomimetic Drugs, Peking University, Beijing, China

**Keywords:** transarterial chemoembolization, multikinase inhibitor, combination therapy, unresectable hepatocellular carcinoma, meta-analysis

## Abstract

**Background:**

Randomized controlled trials (RCTs) testing the combination therapy of transarterial chemoembolization (TACE) plus multikinase inhibitor (MKI) in patients with unresectable hepatocellular carcinoma (HCC) have yielded inconsistent results.

**Methods:**

In this work, a systematic review and meta-analysis was performed to compare the TACE+MKI combination therapy versus TACE monotherapy in HCC patients with time to progression (TTP) adopted as primary outcome.

**Results:**

A total of 10 RCTs comprising 2837 patients receiving combination therapy (TACE plus sorafenib, brivanib, orantinib or apatinib) were included. TACE+MKI significantly prolonged TTP (hazard ratio [HR] 0.74, 95% CI 0.62-0.89, p=0.001) versus TACE monotherapy. Subgroup analysis suggested MKI administration before TACE might be preferable to post-TACE MKI for TTP. TACE+MKI also increased objective response rate (ORR) (risk ratio [RR] 1.17, 95% CI 1.03-1.32, p=0.01), but failed to improve overall survival (OS) (HR 0.98, 95% CI 0.86-1.13, p=0.82) and progression-free survival (PFS) (HR 0.75, 95% CI 0.50-1.12, p=0.16). The incidence of any adverse event (AE) did not significantly differ between TACE+MKI and TACE groups (RR 1.17, 95% CI 0.96-1.42, p=0.01), while serious AEs showed significant difference (RR 1.41, 95% CI 1.26-1.59, p<0.0001). Nevertheless, these AEs showing significant difference were mainly associated with MKI toxicities rather than TACE.

**Conclusions:**

TACE+MKI combination therapy improved TTP and ORR but not OS and PFS in patients with unresectable HCC. Further high-quality trials are needed to verify these clinical benefits, and our findings could be very informative for future trial design.

## 1 Introduction

Liver cancer is the fourth leading cause of cancer-related death worldwide (1), and estimated to affect >1 million individuals annually by 2025 (2). Hepatocellular carcinoma (HCC) is the most common form of liver cancer, and also the most lethal liver tumor with only 18% 5-year survival rate (1). Many etiologies contribute to the development of HCC, with viral hepatitis serving as the most prominent risk factor in the past. However, another pandemic is challenging its position due to effective viral treatment nowadays, for instance, the increasing incidence of NASH makes it already the fastest growing etiology of HCC [Rinaldi, 2021 #6300]. Currently, several treatment options have been adopted as standards of management for patients at different tumour stages, according to clinical practice guidelines (3–5). In principle, early-stage tumours are preferred candidates for liver transplantation, surgical resection or local ablation. Intermediate- stage tumours are potentially treatable by transarterial chemoembolization (TACE), whereas systemic therapy (i.e., sorafenib, atezolizumab plus bevacizumab) represents the mainstream for advanced HCC. All these therapies have contribute to a substantial increase in life expectancy (4–7). However, the overall prognosis remains dismal, owing to the preclusion of early diagnosis and curative treatment (8).

The assignment of TACE in intermediate-stage HCC is based on the evidence from two randomised controlled trials (RCTs) and a subsequent meta-analysis (9–11). Specifically, TACE is preferred to HCC patients at Barcelona Clinic Liver Cancer (BCLC) stage B, defined as being asymptomatic and liver-confined, without portal vein occlusion/thrombosis or extrahepatic spread, namely Child–Pugh class A or class B (5, 12). TACE can concentrate chemotherapeutic agents at the tumour site with higher concentrations than systemic chemotherapy, thus blocking the primary artery feeding the tumour. However, it increases tumour hypoxia, leading to the upregulation of hypoxia inducible factor-1α (HIF-1α), vascular endothelial growth factor (VEGF) and platelet-derived growth factor (PDGF), and thus the increase of tumour angiogenesis, which are associated with a higher risk of extra-hepatic metastasis (13–15). Therefore, it has been proposed that combination therapy of TACE and anti-angiogenic agents should reduce tumour volume and vessel density, and thus improve clinical outcomes.

A number of multikinase inhibitors (MKIs) have been developed for systemic treatment of advanced HCC, since the first approval of sorafenib as first-line treatment. Sorafenib can inhibit a number of serine/threonine and tyrosine kinases (i.e., VEGFR, PDGFR), thereby exerting both anti-angiogenic and direct antitumour effects (16–18). Afterward, lenvatinib, a MKI against VEGFR and FGFR family, is demonstrated non-inferior to sorafenib in terms of overall survival (OS), and then approved for advanced HCC in the first-line setting (19). Additionally, many other oral MKIs [i.e., Brivanib (20), Orantinib (21), Apatinib (22)] showed preliminary efficacy and good safety profile for advanced HCC, whereas their roles in clinical practice have not been established yet.

Both TACE and MKI have been shown to improve survival, and meanwhile MKI in turn may lead to blockade of pro-angiogenic factors induced by TACE. As such, the rationale is clear to combine TACE with MKIs to improve clinical outcomes through inhibiting both tumour proliferation and revascularisation. Several small trials have shown that this combination is effective and safe in patients with unresectable HCC (23, 24). In contrast, most of RCTs testing this combination have failed to show clinical benefits (25–29). Nonetheless, a very recent phase III trial suggested that TACE plus sorafenib significantly improved progression-free survival (PFS) and time to progression (TTP), versus TACE alone (30). Hence, trials assessing the potential synergies between TACE and MKI have yielded inconsistent results. This systematic review and meta-analysis aimed to analyze the efficacy and safety of TACE/MKI combination as compared with TACE alone in patients with unresectable HCC.

## 2 Methods

We followed the PRISMA (Preferred Reporting Items for Systematic Reviews and Meta-analyses) guidelines and checklist for conducting this systematic review (31). The selection criteria regarding target population and outcomes was referenced to AASLD criteria for trial design and end points consensus conference (3). The project was prospectively registered at International Prospective Register of Systematic Reviews (PROSPERO No. CRD42022347259).

### 2.1 Search strategy

The systematic search was conducted using PubMed, EMbase, the Cochrane Library, and Web of Science to capture relevant studies from inception to 18 Nov 2022, without language restriction. Combinations of the following keywords: hepatocellular carcinoma/HCC/liver cancer, sorafenib/lenvatinib/apatinib/sunitinib/axitinib/regorafenib/cabozantinib/donafenib/orantinib/brivanib/tyrosine kinase inhibitor/TKI/multikinase inhibitor/multi-kinase inhibitor/MKI, and chemoembolization/transarterial chemoembolization/TACE, were used in search (see details in **Supplementary Table 1**). The search strategy was designed and conducted by the authors (H.D, T.Y.W, Z.L).

### 2.2 Selection criteria

The records were independently assessed by the authors (H.D, T.Y.W, Z.L) based on the title/abstract and then full-text. Any disagreement between the authors was resolved by discussion to reach a consensus. Studies meeting the following inclusion criteria were considered of eligibility for meta-analysis: 1) trials were described as RCTs; 2) study patients were diagnosed with unresectable HCC, regardless of the kind of treatment they have experienced before; 3) trials comparing at least two different intervention arms (TACE plus MKI versus TACE alone); 4) one of the following outcomes must be included in each trial: TTP, OS, PFS, or objective response rate (ORR). We excluded studies that included participants with pregnancy or breastfeeding. We excluded studies with un-obtainable and unusable data.

### 2.3 Data extraction and quality assessment

The baseline characteristics and outcomes from eligible studies were independently extracted by the authors (H.D, T.Y.W, D.F.G) using a uniform extraction form. Study data included first author, year of publication, sample size, ECOG-PS (Eastern Cooperative Oncology Group performance status), BCLC (Barcelona Clinic Liver Cancer) stage, Child-Pugh score, etiology, follow-up, description of interventions, and type of outcomes (efficacy and safety).

Efficacy outcomes included OS, TTP and PFS, described as hazard ratio (HR) with 95% confidence interval (CI), and ORR. Safety outcomes included patients reporting any adverse event (AE), serious AEs, AE leading to dose interruption, and AE leading to treatment abort. Any disagreement between investigators was resolved by discussion. The risk of bias in the individual studies was assessed using the Cochrane risk of bias tool (32).

### 2.4 Statistical analysis

The primary outcome was TTP, and the secondary outcomes were OS, PFS, ORR and AEs. Meta-analysis was conducted using STATA 16.0 (Stata Corp LLC, TX, USA) using a random-effects model. The pooled HR with 95% confidence interval (CI) was calculated for time-to-event outcomes (TTP, OS and PFS) while pooled risk ratio (RR) was calculated for dichotomous data (ORR and AEs). Subgroup analyses were performed for efficacy outcomes based on the differences in the sequence of TACE and MKI administration. Sensitivity analysis was performed by excluding the study with significant heterogeneity if needed. Heterogeneity was assessed through I^2^ statistic, with values over 50% indicating substantial heterogeneity. Publication bias was not evaluated as the number of studies included in the meta-analysis was too small.

## 3 Results

### 3.1 Study selection, characteristics and quality

**Figure 1:**
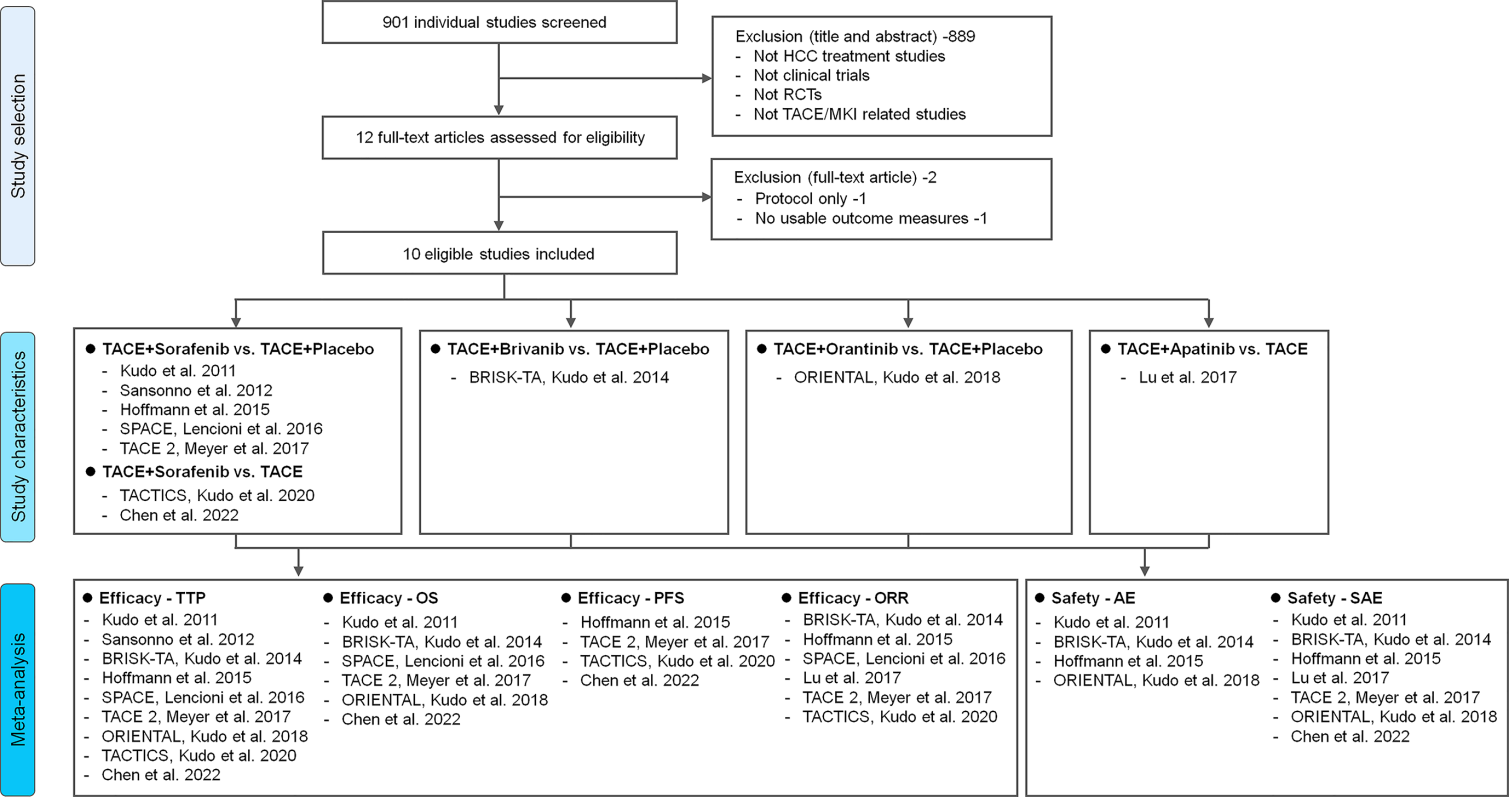
Study flowchart. Description of reasons for including/excluding studies from the current systematic review. Ten RCTs were finally included and four kinds of intervention pairs were compared in these trials (TACE+Sorafenib vs. TACE+Placebo/TACE, TACE+Brivanib vs. TACE+Placebo, TACE+Orantinib vs. TACE+Placebo, TACE+Apatinib vs. TACE). Meta-analysis of different efficacy outcomes (TTP, OS, PFS and ORR) and safety outcomes (AE and SAE) were conducted respectively. HCC, hepatocellular carcinoma; TTP, time to progression; OS, overall survival; PFS, progression-free survival; ORR, objective response rate; AE, adverse event; SAE, serious adverse event; RCT, randomized controlled trial; TACE, transarterial chemoembolization; MKI, multikinase inhibitor.

Overall, a total of 901 unique studies were captured after deleting duplicates, of which 12 were identified as potentially relevant trials (**Figure 1**). After removing two ineligible studies, 10 reports were included for meta-analysis. The detail on fundamental characteristics of included RCTs was summarized in **Supplementary Table 2**. 10 trials included 2837 patients, with 1419 patients treated with TACE+MKI and 1418 treated with TACE+placebo or TACE alone (24–30, 33–35). At baseline, most patients had an ECOG PS of 0; the most common BCLC stage was B (intermediated stage), and most patients had a Child-Pugh Class of A. The etiologies varied across the studies, in which hepatitis B virus (HBV), hepatitis C virus (HCV) and alcohol dominated.

Among the 10 RCTs, four kinds of MKI were respectively combined with TACE (**Figure 1**), in which sorfenib was used in seven trials (25–27, 30, 33–35), while brivanib in BRISK-TA trial (28), orantinib in ORIENTAL trial (29), apatinib in the trial of Lu et al. (24). The detail on intervention characteristics and outcomes was summarized in **Table 1**. There were differences in the phase of trials, agent used, intervention program, follow-up, MKI dosage and primary endpoint across trials. Most of included studies were phase III, multi-centre trials [i.e. TACE 2 (26), BRISK-TA (28), ORIENTAL (29), TACTICS (30)]. The sequence and interval between drug administration and TACE performing varied, for instance, only three trials arranged MKI administration several days/weeks before first TACE [TACE 2 (26), SPACE (27) and TACTICS (30)], while other trials scheduled the first TACE session before MKI initiation. The median follow-up was specified in eight trials, ranging from the minimum 9.0 months in SPACE (27) to maximum 30.6 months in TACTICS (30). Among trials reporting the median dose or period of MKI, the maximum median dose of MKI (sorafenib, 660 mg) was shown in TACE 2 trial (26), while the maximum period of drug therapy (orantinib, 10.9 months) was shown in ORIENTAL trial (29). Regarding endpoints, TTP was adopted as the primary endpoint in four trials (25, 27, 33, 34), OS in two trials (28, 29), PFS in one trial (26), OS and PFS as the co-primary endpoints in one trial (30), ORR in one trial (24) and time-to-complete response in one trial (35).

**Table 1:** Intervention characteristics and study outcome measures of included studies in meta-analysis.

Study	Intervention	Patients included	Follow-up (mo)	MKI dose	Median dose & period (mo)	TTP (mo)		OS (mo)		PFS (mo)		ORR, n (%)
						Median	HR (95% CI)	Median	HR (95% CI)	Median	HR (95% CI)	
Kudo et al. 2011 (Eur J Cancer)	TACE first, then sorafenib	229	NA	400 mg twice daily	386 mg & 4.3	5.4	0.87 (0.70-1.09)	29.7	1.06 (0.69-1.64)	NA	NA	NA
	TACE first, then placebo	229	NA		786 mg & 5.0	3.7		NE		NA		NA
Sansonno et al. 2012 (Oncologist)	TACE first, sorafenib initiates 30 days after TACE	31	NA	400 mg twice daily	NA	9.2	0.40 (0.27-0.60)	NA	NA	NA	NA	NA
	TACE first, placebo initiates 30 days after TACE	31	NA		NA	4.9		NA		NA		NA
Kudo et al. 2014 (BRISK-TA, Hepatol)	TACE first, then brivanib no less than 48 hours, but no longer than 21 days after TACE	249	16.6	800 mg once-daily	NA & 6.0	12.0	0.94 (0.72-1.22)	26.4	0.90 (0.66-1.23)	NA	NA	120 (48)
	TACE first, then placebo	253	15.6		NA	10.9		26.1		NA		106 (42)
Hoffmann et al. 2015 (BMC Cancer)	TACE first, sorafenib was given 3 days before and after each TACE	24	10.7	400 mg twice daily	NA & 4.2	2.4	1.11 (0.39-3.16)	NA	NA	NA	1.26 (0.49-3.27)	5 (21)
	TACE first, then placebo	26			NA & 5.7	2.8		NA		NA		7 (27)
Lencioni et al. 2016 (SPACE, J Hepatol)	Sorafenib 3-7 days before first TACE	154	9.0	400 mg twice daily	566 mg & 5.3	5.6	0.80 (0.59-1.08)	NE	0.90 (0.61-1.33)	NA	NA	55 (36)
	Placebo 3-7 days before first TACE	153	9.1		791 mg & 6.8	5.5		NE		NA		43 (28)
Lu et al. 2017 (Cancer Biol Ther)	TACE first, then apatinib 4 days after TACE	20	9.7	500 mg/day	NA	NA	NA	NA	NA	12.5	NA	7 (35)
	TACE alone	22			NA	NA		NA		6.0		2 (9)
Meyer et al. 2017 (TACE 2, Lancet Gastroenterol Hepatol)	Sorafenib 2-5 weeks before first TACE	157	20.7	400 mg twice-daily	660 mg & 4.0	10.9	0.88 (0.67-1.17)	21.0	0.91 (0.67-1.24)	34.0	0.99 (0.77-1.27)	56 (36)
	Placebo 2-5 weeks before first TACE	156			800 mg & 5.4	10.7		19.9		33.6		49 (31)
Kudo et al. 2018 (ORIENTAL, Lancet Gastroenterol Hepatol)	TACE first, then orantinib between days 3 and 28 after the first (and any subsequent) TACE	444	17.3	200 mg twice daily	NA & 10.9	2.9	0.86 (0.74-0.99)	31.1	1.09 (0.88-1.35)	NA	NA	NA
	TACE first, then placebo	444			NA & 12.3	2.5		32.3		NA		NA
Kudo et al. 2020 (TACTICS, Gut)	Sorafenib 2-3 weeks before first TACE	80	30.6	400 mg once daily before TACE, 800 mg once daily during TACE sessions	355 mg & 9.7	26.7	0.54 (0.35-0.83)	NA	NA	25.2	0.59 (0.41-0.87)	57 (71)
	TACE alone	76			NA	16.4		NA		13.5		47 (62)
Chen et al. 2022 (Hepatol Int)	TACE first, sorafenib was given 3 days before and after each TACE	29	23.8	400 mg/day	NA & 5.2	32.2	0.37 (0.18-0.77)	NE	0.68 (0.21-2.16)	24.8	0.46 (0.24-0.90)	NA
	TACE alone	30	NA		NA	14.5		31.0		14.5		NA

mo, months; TTP, time to progression; OS, overall survival; PFS, progression-free survival; ORR, objective response rate; TACE, transarterial chemoembolization; HR, hazard ratio; CI, confidence interval; NA, not available; NE, not estimable because of immaturity of data.

Data are n (%) for categories, and median for continuous data.

Risk of bias assessment of included trials was based on the Cochrane risk of bias tool, and the detail was presented in **Supplementary Figure 1**. The risk of bias was generally low across trials. Specifically, all trials showed no risk of selection bias. However, three trials were open-label with potential risk of performance bias and detection bias (24, 30, 35), while others claimed double-blinded.

### 3.2 Efficacy outcomes

#### 3.2.1 TTP

Our systematic review identified nine trials reporting TTP as either primary or secondary endpoint. Among them, seven trials evaluating TACE/sorafenib combination versus TACE alone. A meta-analysis with inclusion of these seven trials was conducted, and found significantly prolonged TTP in TACE+Sorafenib group versus TACE group (HR 0.67, 95% CI 0.52-0.87, p=0.003) (**Supplementary Figure 2**). Given that four trials scheduled sorafenib administration after TACE session while another three trials designed sorafenib plus following TACE, subgroup analyzes were conducted based on the administration sequence between sorafenib and TACE. The pooled results of four trials showed no significant difference between groups (HR 0.60, 95% CI 0.34-1.03, p=0.07), suggesting no clinical benefit of TTP from TACE combined with following sorafenib versus TACE alone. In contrast, the pooled results of another three trials demonstrated a significant difference in TTP between groups (HR 0.76, 95% CI 0.59-0.97, p=0.03). Therefore, the combination of sorafenib with TACE could improve TTP, and sorafenib administration prior TACE could be superior to that after TACE, in terms of TTP.

**Figure 2:**
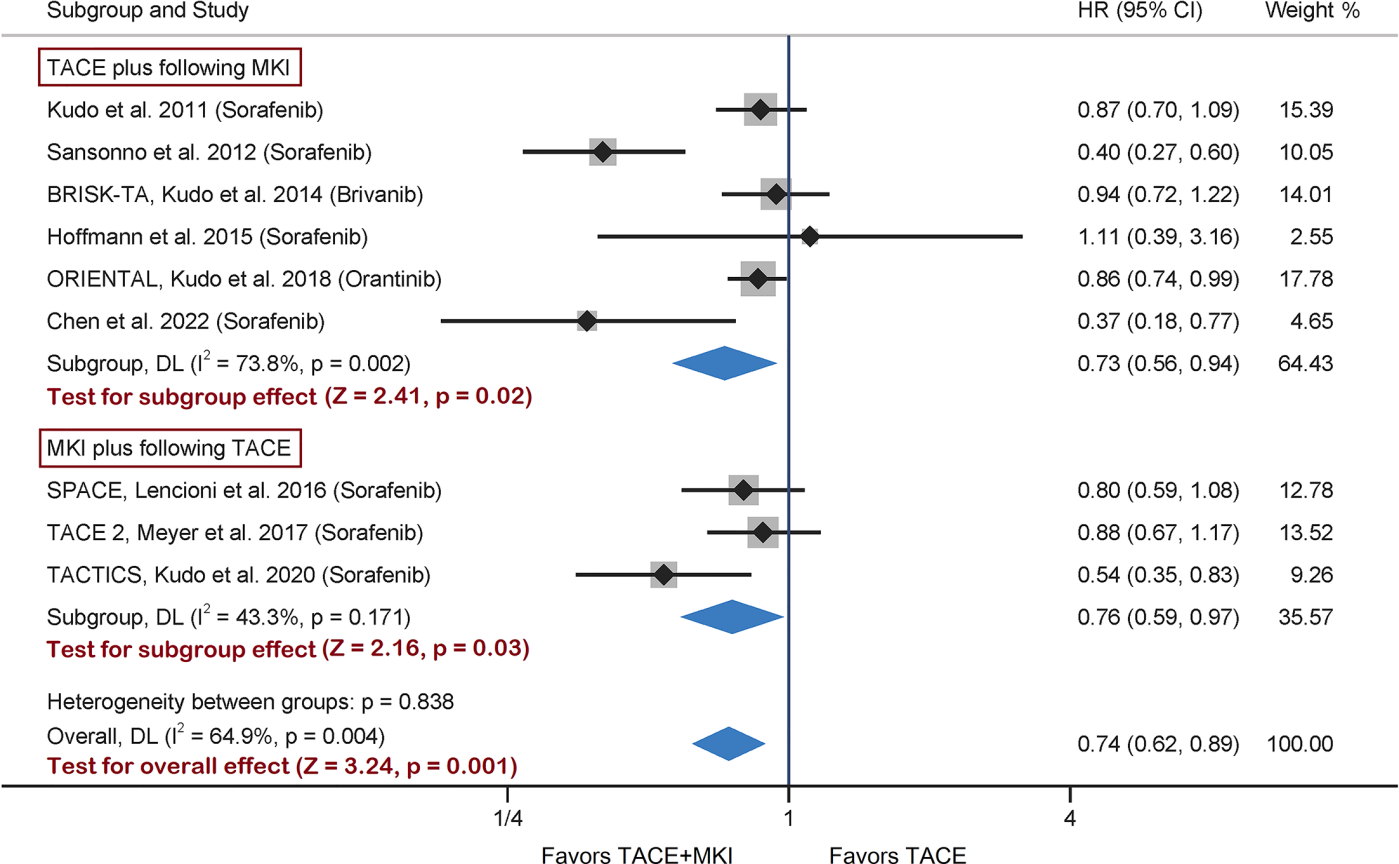
Meta-analysis of treatment effects of MKI in combination with TACE on TTP in patients with unresectable HCC. For subgroups analysis, 9 trials are divided into two classes based on TACE schedule: TACE plus following MKI & MKI plus following TACE. The MKI evaluated in these trials included sorafenib, brivanib and orantinib. The pooled HR of TTP was calculated by using a random-effects model, and the variance of the distribution of true effect sizes was estimated by means of the DerSimonian-Laird method. MKI, multikinase inhibitor; TACE, transarterial chemoembolization; TTP, time to progression; HR, hazard ratio.

The remaining two trials scheduled brivanib or orantinib administration after TACE session (28, 29). A meta-analysis taking all nine trials together was conducted, and found a significant difference in TTP between TACE+MKI group and TACE group (HR 0.74, 95% CI 0.62-0.89, p=0.001) (**Figure 2**). This result was consistent with the pooled result above from seven sorafenib-trials, although the effect size differ slightly but not significantly. Besides, subgroup analyzes by prior or post TACE were consistent with the overall findings (HR 0.73, 95% CI 0.56-0.94, p=0.02; HR 0.76, 95% CI 0.59-0.97, p=0.03, respectively). In view of high heterogeneity across studies, a sensitivity analysis was conducted by removing the study of Sansonno et al. which was the source of heterogeneity, and the overall results were almost identical (HR 0.82, 95% CI 0.72-0.93, p=0.003) (**Supplementary Figure 3**). Based on these results, the combination of TACE with MKI could improve TTP versus TACE alone, and scheduling MKI administration before TACE might be superior to that after TACE.

#### 3.2.2 OS

**Figure 3:**
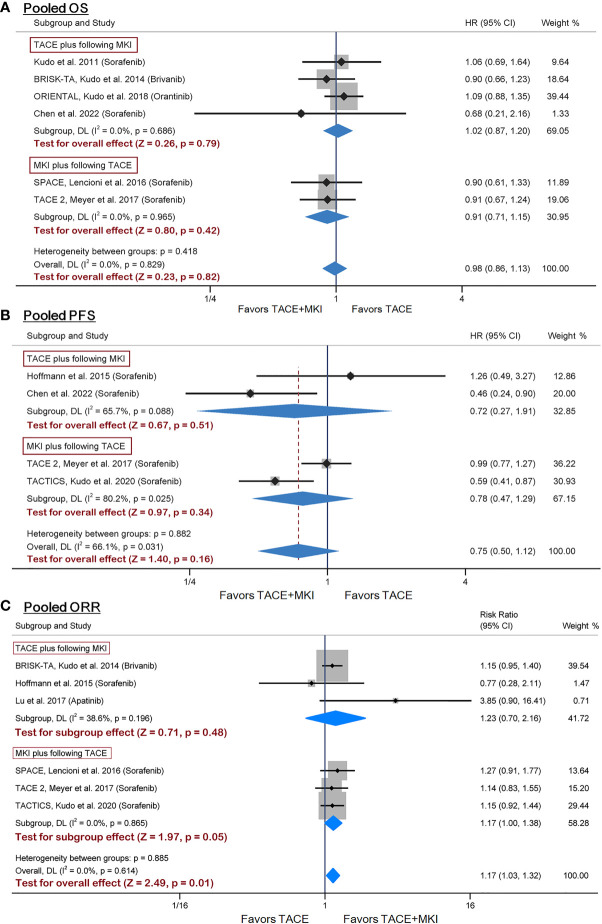
Meta-analysis of treatment effects of MKI in combination with TACE on OS **(A)**, PFS **(B)** and ORR **(C)** in patients with unresectable HCC. For subgroups analysis, trials are divided into two classes based on TACE schedule: TACE plus following MKI & MKI plus following TACE. The MKI evaluated in these trials included sorafenib, brivanib and orantinib. The pooled HR or RR was calculated by using a random-effects model, and the variance of the distribution of true effect sizes was estimated by means of the DerSimonian-Laird method. MKI, multikinase inhibitor; TACE, transarterial chemoembolization; OS, overall survival; PFS, progression- free survival; ORR, objective response rate; HR, hazard ratio; RR, risk ratio.

Six of ten RCTs adopted OS as an endpoint (25–29, 35). Besides, sorafenib was administrated to patients in four trials while brivanib in BRISK-TA trial (28), and orantinib in ORIENTAL trial (29). Given the diversity of administrated drugs, we firstly performed a meta-analysis on the four trials evaluating the combination of sorafenib with TACE, and found no significant difference in OS between groups (**Supplementary Figure 4**). Subsequently, a meta-analysis integrating all six trials showed consistent results (HR 0.98, 95% CI 0.86-1.13, p=0.82) (**Figure 3A**). Besides, subgroup analysis found no difference in the effect sizes between the subgroup adopting prior TACE and that adopting post TACE (**Figure 3A**). Therefore, these results suggested that the combination of sorafenib with prior or post TACE failed to yield superior OS to TACE alone.

#### 3.2.3 PFS

Four trials reporting PFS as an endpoint all designed sorafenib administration as adjuvant therapy to TACE (26, 30, 34, 35). We performed a meta-analysis on these four trials with inclusion of a total of 578 patients, demonstrating no significant difference in PFS between groups (HR 0.75, 95% CI 0.50-1.12, p=0.16) (**Figure 3B**). Additionally, subgroup analysis was conducted to examine whether the sequence between sorafenib administration and TACE operation may have affected PFS. Results by TACE plus following sorafenib or sorafenib plus following TACE were consistent with the overall findings (**Figure 3B**). Based on these results, the combination of sorafenib with TACE failed to yield superior PFS to TACE alone.

#### 3.2.4 ORR

We identified six trials reporting ORR for inclusion into meta-analysis (24, 26–28, 30, 34). The ORR in sorafenib group ranged from 20.8% to 71.3% across trials. The pooled results of meta-analysis found that combination therapy significantly increased ORR versus TACE mono-therapy (risk ratio 1.17, 95% CI 1.03-1.32, p=0.01), although no significant difference were found from subgroup analysis (**Figure 3C**). The sensitivity analysis by using odds ratio as summary statistic yielded consistent results (odds ratio 1.33, 95% CI 1.07-1.67, p=0.012) (**Supplementary Figure 5**). The above results demonstrated that the combination of TACE and MKI could improve ORR, compared with TACE alone.

### 3.3 Safety outcomes

**Figure 4:**
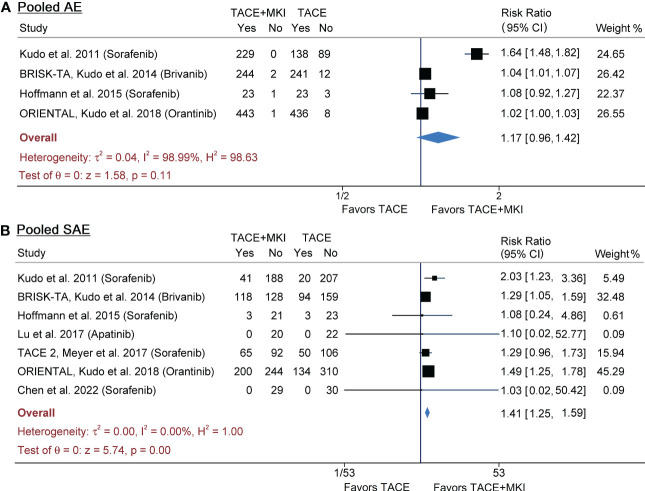
Meta-analysis of AEs **(A)** and SAEs **(B)** of MKI in combination with TACE in patients with unresectable HCC. The MKI evaluated in these trials included sorafenib, brivanib and apatinib. The pooled risk ratio was calculated by using a random-effects model. AE, adverse event; SAE, serious adverse event; MKI, multikinase inhibitor; TACE, transarterial chemoembolization.

**Table 1** summarized the AEs, AE leading dose interruption and AE leading treatment abort in either group of included studies. The pooled results from meta-analysis demonstrated that the incidence of any AE was not significantly different (risk ratio 1.17, 95% CI 0.96-1.42, p=0.11), although it was slightly lower in TACE group than in TACE+MKI group in all 4 trials (**Figure 4A**). Across the seven trials reporting serious AEs, their incidence varied strikingly (i.e., 0 to 48% in TACE+MKI groups) (24–26, 28, 29, 34, 35). Meta-analysis demonstrated that the incidence of serious AEs was significantly higher in patients receiving TACE+MKI than that receiving TACE alone (risk ratio 1.41, 95% CI 1.25-1.59, p<0.0001) (**Figure 4B**).

The most frequent, treatment-emergent AEs were abdominal pain, hand-foot skin reaction (HFSR), fatigue, pyrexia, anorexia, diarrhea, hypertension and thrombocytopenia in either group across studies. The incidence of these AEs in each trial was summarized in **Table 2**. The distribution and weighted means of incidence of each AE across trials were provided in **Supplementary Figure 4**. They were ranked as abdominal pain > HFSR > fatigue > pyrexia > anorexia > diarrhea > hypertension > thrombocytopenia in TACE+MKI group, while abdominal pain > pyrexia > fatigue > anorexia > diarrhea > thrombocytopenia > hypertension > HFSR in TACE group, according to the weighted means of incidence. Additionally, meta-analysis was performed on each kind of AEs to explore their differences between groups (**Supplementary Figure 6**). The pooled results demonstrated that the incidence of HFSR, fatigue, anorexia, diarrhea, hypertension and thrombocytopenia was significantly higher in TACE+MKI group than TACE group, respectively, while there was no significant difference in the incidence of abdominal pain and pyrexia between groups (**Supplementary Figures 6, 7**).

**Table 2:** Summary of adverse events in either group of included studies in meta-analysis.

Study	Intervention	Patients included	AE, n (%)																	AE→ dose interruption	AE→ treatment abort
			Any	Grade ≥3	Serious AE	Abdominal pain		HFSR	Fatigue		Pyrexia		Anorexia		Diarrhoea		Hypertension		Thrombocytopenia		
Kudo et al. 2011	TACE+Sorafenib	229	229 (100)	NA	41 (18)	29 (13)		188 (82)	46 (20)		37 (16)		45 (20)		71 (31)		71 (31)		57 (25)	163 (71)	93 (41)
	TACE+Placebo	227	138 (61)	NA	20 (9)	21 (9)		16 (7)	34 (15)		25 (11)		18 (8)		11 (5)		16 (7)		5 (2)	27 (12)	13 (6)
Sansonno et al. 2012	TACE+Sorafenib	40	NA	NA	NA	NA		4 (10)	9 (23)		NA		3 (8)		4 (10)		6 (15)		NA	8 (5)	9 (22)
	TACE+Placebo	40	NA	NA	NA	NA		0 (0)	3 (8)		NA		4 (10)		3 (8)		4 (10)		NA	0 (0)	0 (0)
Kudo et al. 2014 (BRISK-TA)	TACE+Brivanib	246	244 (>99)	172 (69)	118 (48)	90 (37)		77 (31)	101 (41)		93 (38)		106 (43)		88 (36)		116 (47)		58 (24)	68 (28)	98 (40)
	TACE+Placebo	253	241 (95)	109 (43)	94 (37)	101 (40)		5 (2)	59 (23)		115 (46)		57 (23)		25 (10)		29 (11)		42 (17)	7 (3)	46 (18)
Hoffmann et al. 2015	TACE+Sorafenib	24	23 (92)	12 (50)	3 (13)	0 (0)		7 (29)	5 (21)		0 (0)		0 (0)		9 (38)		0 (0)		13 (54)	6 (25)	6 (25)
	TACE+Placebo	26	23 (96)	4 (16)	3 (12)	0 (0)		1 (4)	5 (21)		0 (0)		0 (0)		3 (12)		0 (0)		14 (56)	2 (8)	1 (4)
Lencioni et al. 2016 (SPACE)	TACE+Sorafenib	153	NA	NA	NA	92 (60)		71 (46)	66 (43)		59 (39)		47 (31)		81 (53)		46 (30)		NA	133 (87)	129 (84)
	TACE+Placebo	151	NA	NA	NA	93 (62)		10 (7)	50 (33)		52 (34)		31 (21)		26 (17)		25 (17)		NA	93 (61)	63 (42)
Lu et al. 2017	TACE+Apatinib	20	NA	3 (15)	0 (0)	9 (45)		11 (55)	NA		15 (75)		NA		4 (20)		16 (80)		NA	NA	3 (15)
	TACE	22	NA	0 (0)	0 (0)	12 (55)		0 (0)	NA		17 (77)		NA		1 (5)		1 (5)		NA	NA	0 (0)
Meyer et al. 2017 (TACE 2)	TACE+Sorafenib	157	NA	NA	65 (41)	93 (59)		65 (41)	127 (81)		NA		53 (34)		87 (55)		NA		NA	NA	30 (19)
	TACE+Placebo	156	NA	NA	50 (32)	89 (57)		13 (8)	122 (78)		NA		52 (33)		49 (31)		NA		NA	NA	16 (10)
Kudo et al. 2018 (ORIENTAL)	TACE+Orantinib	444	443 (>99)	NA	200 (45)	317 (71)		43 (10)	101 (23)		264 (59)		209 (47)		123 (28)		59 (13)		53 (12)	160 (36)	96 (22)
	TACE+Placebo	444	436 (98)	NA	134 (30)	292 (66)		51 (11)	92 (21)		284 (64)		149 (34)		70 (16)		57 (13)		48 (11)	37 (8)	49 (11)
Kudo et al. 2020 (TACTICS)	TACE+Sorafenib	77	NA	NA	NA	NA		41 (53)	19 (25)		15 (20)		11 (14)		11 (14)		40 (52)		67 (87)	77 (100)	2 (3)
	TACE	71	NA	NA	NA	NA		0 (0)	7 (10)		18 (25)		8 (11)		0 (0)		28 (39)		53 (75)	NA	2 (3)
Chen et al. 2022	TACE+Sorafenib	29	NA	5 (26)	0 (0)	0 (0)		17 (59)	6 (21)		14 (48)		2 (7)		6 (21)		3 (10)		12 (41)	0 (0)	0 (0)
	TACE	30	NA	0 (0)	0 (0)	0 (0)		0 (0)	3 (10)		11 (37)		3 (10)		0 (0)		1 (3)		7 (23)	0 (0)	0 (0)

AE, adverse event; HFSR, Hand- foot skin reaction; TACE, transarterial chemoembolization; NA, not available.

## 4 Discussion

As a kind of local therapeutic-strategy, TACE has become the standard of care for patients with intermediate stage HCC. However, the repetition of TACE results in two major problems: deteriorated liver function and increased tumour angiogenesis (13–15). The tumour angiogenesis is attributed to the acute hypoxia caused by TACE which consequently leads to the upregulation of some kinases, such as VEGF and PDGF. As such, it seems promising to schedule MKI administration as adjuvant therapy to TACE to improve clinical outcomes with the assistance of its both antiangiogenic and direct antitumour effects. To date, many RCTs have evaluated the combination of TACE with MKI (i.e. sorafenib, brivanib, orantinib and apatinib) in patients with unresectable HCC, however, yielded inconsistent results (24–30, 33–35).

Several meta-analyses have been done on TACE/sorafenib combination. The meta-analysis of Wang et al. (36) included five comparative studies (only two RCTs) found that TACE+sorafenib improved TTP (HR 0.61, 95% CI 0.39-0.95; pooled result of three studies) but failed to improve OS (HR 0.79, 95% CI 0.54-1.16; pooled result of three studies). However, their findings were limited by the small number of included studies, high heterogeneity across the studies, and especially the mixed RCTs, prospective and retrospective studies. Two subsequent network meta-analyses respectively included 5 and 6 trials to compare TACE+sorafenib versus TACE (37, 38). Whereas, some of these included trials they claimed RCTs turned out to be nonrandomized which drastically challenged the credibility of their findings. A latest network meta-analysis conducted by Zhang et al. (39) found that TACE plus TKIs (apatinib, lenvatinib, or sorafenib) significantly benefited OS (HR 2.09, 95% CI 1.50-2.91; HR 2.72, 95% CI 1.37-5.59; and HR 1.46, 95% CI 1.20-1.75, respectively) and PFS (HR 1.67, 95% CI 1.12-2.63; HR 2.99, 95% CI 1.72-5.28; and HR 1.54, 95% CI 1.17-2.08, respectively), compared with TACE monotherapy. However, only five trials were RCTs while others were cohort studies among the included 41 studies. Additionally, the outcome measures of TTP and AEs were not evaluated in this meta-analysis.

This meta-analysis provides currently the most comprehensive synthesis of comparative data from RCTs on the efficacy and safety of TACE/MKI combination versus TACE. We found that MKI as adjuvant therapy to TACE improved TTP and ORR, but not OS or PFS. Specifically, the meta-analysis with inclusion of nine trials found that TACE+MKI significantly prolonged TTP versus TACE alone, and subgroup analysis by prior or post TACE yielded consistent results. Besides, sensitivity analysis by removing the heterogenous study found that TTP did not differ significantly in subgroup of TACE plus following MKI while it differ significantly in subgroup of MKI plus following TACE. In regard to sorafenib, the meta-analysis of seven RCTs demonstrated that combination therapy significantly increased TTP, and subgroup analysis suggested that sorafenib administration prior to TACE could be superior to that after TACE regarding TTP. Likewise, Overall, the combination of TACE with MKI could result in longer TTP than TACE alone in patients with unresectable HCC, and scheduling MKI administration before TACE session might be superior to that after TACE operation.

MKI administration is designed to suppress tumour angiogenesis induced by TACE, thus timing for drug administration, relative to TACE, represents a key to maximize its efficacy. The ORIENTAL trial provided a clue that patients in orantinib group with a VEGF-C concentration below the median value showed significantly prolonged time to TACE failures (29), indicating that low VEGF level might contribute to a favourable clinical outcome. Since serum VEGF reaches maximum concentration on day 1 after TACE (13), MKI may exert the greatest effects when administered immediately after or even before TACE. Correspondingly, SPACE trial firstly tested the efficacy of sorafenib plus following TACE in which sorafenib was administrated 3-7 days before the first TACE (27). In particular, this combination improved TTP according to the predefined statistical threshold (HR 0.79, one-sided p= 0.072), despite no difference in median TTP between groups (27). The subsequent TACE 2 with similar study design yet failed to provide positive result for TTP (26). In contrast, the latest TACTICS trial reported a significantly longer TTP in TACE+Sorafenib group than TACE group (26.7 vs. 20.6 months, p=0.02) and also a significantly longer PFS (25.2 vs. 13.5 months, p=0.006) (30). These favourable outcomes may be due to pre-treatment with sorafenib 2-3 weeks before the initial TACE, as well as the long median duration of sorafenib treatment (38.7 weeks). To sum up, MKI administration before TACE could be preferable to post-TACE MKI for prolonging TTP, which was evidenced by our findings. However, the optimal timing for MKI administration has not reach a consensus, thus further high-quality trials are needed to verify the clinical benefit from MKI pre-treatment combined with TACE.

There has been no consensus regarding the primary endpoints in TACE/MKI combination trials. OS is objective and clinically relevant serving as the sole robust endpoint. The phase III trials BRISK-TA and ORIENTAL chose OS as the primary endpoint (28, 29). However, OS measure requires long follow-up time to capture the events (40), thus being a critical limitation when evaluating interventions for HCC at early or intermediate stages. Additionally, in particular of MKI/TACE combination trial, the high rate of crossover to MKI might obscure any benefit of the combination if OS is adopted as the primary endpoint (26). Therefore, several surrogate endpoints, such as PFS, TTP and ORR, have been proposed although lacking of adequate validation. For these endpoints, tumour response is mostly assessed by the Response Evaluation Criteria in Solid Tumors (RECIST) criteria or modified RECIST (mRECIST), but TACTICS trial adopted Response Evaluation Criteria in Cancer of the Liver (RECICL) to define progression. Although TTP has been suggested of weakness for predicting clinically relevant improvement in OS, it indeed has its own strong point in capturing the implied clinical benefit from combination therapy: the differences in TTP are not masked by the second treatment despite the crossover efficacy in combination therapy. Consistently, among the included 10 trials, TTP was chosen as the primary endpoint in four trials (25, 27, 33, 34), and as the secondary endpoint in five trials (26, 28–30, 35). Therefore, TTP was selected as the primary outcome in our meta-analysis.

The combination of TACE with MKI was clinically safe cross trials. The most frequent AEs related to TACE are typical of post-embolization syndrome, such as abdominal pain, pyrexia and nausea (41). Regarding MKI, the most common AEs are HFSR, diarrhoea and hypertension. Although some AEs were more frequently observed in the TACE+MKI group than TACE group, the addition of MKI didn’t seem to increase toxicity associated with TACE. It was evidenced by no significant difference in the incidence of abdominal pain and pyrexia between groups in our meta-analysis. Furthermore, the major differences were related to the well-known toxicities of MKI, as evidenced by that significantly higher incidence of HFSR, fatigue, anorexia, diarrhea, hypertension and thrombocytopenia was found in TACE+MKI group.

Some limitations should be acknowledged in our meta-analysis. First, the number of included studies for meta-analysis (range 4-9 comparative studies) was small. Second, there were differences in the tools for defining tumour progression across trials, for instance, RECIST in TACE 2 trial (26), mRECIST in BRISK-TA trial (28), while RECICL in TACTICS trial (30). Third, specific data for subset analysis by etiology or region were not available among trials, while the effects of regional or etiologic variability on outcomes might be latent. Forth, HCC patients may also present several comorbidities beyond the hepatic problem itself which may alter outcomes, whereas this work failed to evaluate the comorbidity burden due to the varied exclusion criteria across studies and unavailable data. Finally, the trials included were heterogeneous, comprising the differences in study population, clinical characteristics (i.e., ECOG-PS, BCLC stage and Child-Pugh stage), the variety of MKI (sorafenib, brivanib, orantinib and apatinib), as well as the different timings of MKI administration, which might impact the treatment efficacy and cause bias in the pooled results.

## 5 Conclusions

In summary, our meta-analysis found that the combination of TACE with MKI could result in improved TTP and ORR in patients with unresectable HCC versus TACE alone. Besides, pre-treatment with MKI, relative to TACE, might lead to a better outcome of TTP than post-TACE MKI treatment. However, this combination failed to improve OS and PFS. The addition of MKI doesn’t seem to increase toxicity associated with TACE. Some AEs, occurred more frequently in the combination group, were associated with the well-known toxicities of MKI. Despite some limitations in this work, we provide updated and comprehensive evidence on the efficacy and safety of TACE/MKI combination therapy, and our findings could be very informative for future clinical trial design.

## Data availability statement

The original contributions presented in the study are included in the article/**Supplementary Material**. Further inquiries can be directed to the corresponding authors.

## Author contributions

ZL, TW and JW: conceptualization and supervision. ZL and HD: writing. ZL and DG: revision. DG, PZ and BQ: software and methodology, data curation. HD, QW and BQ: investigation and formal analysis. All authors contributed to the article and approved the submitted version.
