# Supplementary material for: Transarterial chemoembolization with or without multikinase inhibitors for patients with unresectable hepatocellular carcinoma: a systematic review and meta-analysis of randomized controlled trials
Source: Front Oncol. 2023 Jun 8;13:1139025. doi: 10.3389/fonc.2023.1139025 (PMC10285094; doi:10.3389/fonc.2023.1139025)
Supplement: Supplementary file 1 [file DataSheet_1.pdf]

Supplementary Material

Transarterial Chemoembolization plus Multikinase Inhibitors for Patients with Unresectable Hepatocellular Carcinoma: A Systematic Review and Meta-analysis of Randomized Controlled Trials

Han Dong [1,†], Dongfang Ge [2,†], Biao Qu [3], Ping Zhu [4], Qibiao Wu [5], Tianyun Wang [4,6,*], Jue Wang [5,*], Zheng Li [7,8,*]

[1]Department of Nursing, Huaian Hospital of Huaian City, Huaian, China

[2]President's Office of Huaian Hospital of Huaian City, Huaian, China

[3]Department of Clinical Pharmacology, The Second Hospital of Anhui Medical University, Hefei, China

[4]Department of Endocrinology, Huaian Hospital of Huaian City, Huaian, China

[5]State Key Laboratory of Quality Research in Chinese Medicines, Faculty of Chinese Medicine, Macau University of Science and Technology, Macau, China

[6]Department of Pharmacy, Huaian Hospital of Huaian City, Huaian, China

[7]College of Health Sciences, School of Life Sciences, Jiangsu Normal University, Xuzhou, China

[8]State Key Laboratory of Natural and Biomimetic Drugs, Peking University, Beijing, China

[†]These authors contributed equally to this work.

***Correspondence:**

Tianyun Wang, Department of Pharmacy, Huaian Hospital of Huaian City, Huaian, China. Email: 15952152603@163.com

Jue Wang, State Key Laboratory of Quality Research in Chinese Medicines, Macau University of Science and Technology, Macau, China. Email: wangjue@must.edu.mo

Zheng Li, College of Health Sciences, School of Life Sciences, Jiangsu Normal University, Xuzhou, China. Email: lizhengcpu@163.com

Supplementary Table 1 Search strategy with PubMed as an example

1	"Carcinoma, Hepatocellular"[MeSH Terms]
2	hepatocellular carcinoma OR HCC[Title/Abstract]
3	liver cancer*[Title/Abstract]
4	cancer of the liver[Title/Abstract]
5	#1 OR #2 OR #3 OR #4
6	tyrosine kinase inhibitor OR TKI[Title/Abstract]
7	multikinase inhibitor* OR multi-kinase inhibitor* OR MKI[Title/Abstract]
8	"Sorafenib"[MeSH Terms]
9	sorafenib OR lenvatinib OR apatinib OR sunitinib OR axitinib OR regorafenib OR cabozantinib OR donafenib OR orantinib OR brivanib[Title/Abstract]
10	#6 OR #7 OR #8 OR #9
11	"Chemoembolization, Therapeutic"[Mesh]
12	chemoembolisation OR transarterial chemoembolization OR TACE[Title/Abstract]
13	#11 OR #12
14	#5 AND #10 AND #13

Supplementary Table 2. Baseline characteristics of included studies in meta-analysis

Study	Study design	Group	Cases	Age (years)	Male, n (%)	ECOG-PS, n (%)		BCLC stage, n (%)					Child-Pugh Class, n (%)			Etiology, n (%)		
						0	1	0	A	B	C	D	A	B	C	HBV	HCV	Alcohol
Kudo et al. 2011	Multi-centre RCT, phase III	TACE+Sorafenib	229	69	174 (76)	201 (88)	28 (12)	NA					NA			139 (60)	47 (20)	19 (8)
		TACE+Placebo	229	70	168 (73)	202 (88)	27 (12)	NA					NA			148 (65)	52 (23)	12 (5)
Sansonno et al. 2012	Single-center RCT	TACE+Sorafenib	31	73 ± 4	18 (58)	26 (76)	5 (24)	NA					31 (100)				31 (100)	
		TACE+Placebo	31	73 ± 6	19 (61)	24 (77)	7 (23)	NA					31 (100)				31 (100)	
Kudo et al. 2014 (BRISK-TA)	Multinational RCT, phase III	TACE+Brivanib	249	57 (21-85)	206 (83)	201 (81)	48 (19)		65 (26)	129 (52)	54 (22)	1 (<1)	239 (96)	9 (4)	1 (<1)	158 (63)	49 (20)	40 (16)
		TACE+Placebo	253	59 (25-85)	216 (85)	203 (80)	50 (20)		57 (23)	150 (59)	44 (17)	2 (1)	231 (91)	20 (8)	2 (1)	168 (66)	42 (17)	38 (15)
Hoffmann et al. 2015	Multi-centre RCT, phase III	TACE+Sorafenib	24	59 (44-66)	45 (90)	NA		NA					14 (58)	9 (38)	1 (4)	3 (13)	11 (46)	7 (29)
		TACE+Placebo	26	58 (43-69)		NA		NA					20 (77)	6 (23)	0 (0)	3 (12)	7 (27)	11 (42)
Lencioni et al. 2016 (SPACE)	Multi-centre RCT, phase II	TACE+Sorafenib	154	65	135 (88)	154 (100)				154 (100)			153 (99)	1 (<1)		55 (36)	39 (25)	27 (18)
		TACE+Placebo	153	63	126 (82)	153 (100)				153 (100)			152 (99)			50 (33)	41 (27)	30 (20)

Lu et al. 2017	Single-center RCT	TACE+Apatinib	22	(39-75)	17 (77)	NA				19 (86)	3 (14)	NA		20 (91)		2 (9)
		TACE	20	(34-79)	16 (80)	NA				18 (90)	2 (10)	NA		18 (90)		2 (10)
Meyer et al. 2017 (TACE 2)	Multi-centre RCT, phase III	TACE+Sorafenib	157	65 (57-71)	139 (89)	98 (62)	58 (37)	NA				145 (93)	5 (4)	7 (5)	15 (12)	44* (34)
		TACE+Placebo	156	68 (63-74)	138 (88)	97 (62)	58 (37)	NA				148 (95)	3 (2)	7 (6)	9 (7)	40 (33)
Kudo et al. 2018 (ORIENTAL)	Multi-centre RCT, phase III	TACE+Orantinib	444	67 (59-75)	363 (82)	401 (90)	43 (10)	10 (2)	148 (33)	209 (47)	74 (17)	444 (100)		170 (38)	193 (43)	
		TACE+Placebo	444	66 (59-73)	364 (82)	406 (91)	38 (9)	13 (3)	122 (27)	229 (52)	72 (16)	444 (100)		202 (45)	165 (37)	
Kudo et al. 2020 (TACTICS)	Multi-centre RCT	TACE+Sorafenib	80	72 (36-85)	63 (79)	NA			27 (34)	44 (55)	9 (11)	79 (99)	1 (1)	10 (13)	38 (48)	
		TACE	76	73 (53-86)	55 (72)	NA			33 (43)	34 (45)	9 (12)	71 (93)	5 (7)	2 (3)	53 (70)	
Chen et al. 2022	Multi-centre RCT	TACE+Sorafenib	29	67 ± 10	24 (83)	27 (93)	2 (7)	NA				22 (76)	7 (24)	15 (52)	9 (31)	13 (45)
		TACE	30	63 ± 14	24 (80)	28 (93)	2 (7)	NA				24 (80)	6 (20)	13 (43)	15 (50)	13 (43)

Abbreviations: ECOG-PS=Eastern Cooperative Oncology Group performance status; BCLC=Barcelona Clinic Liver Cancer; HBV=hepatitis B virus; HCV=hepatitis C virus; TACE=transarterial chemoembolization; RCT=randomised controlled trial; NA=not available.
* Etiology data were available for the patients with cirrhosis.

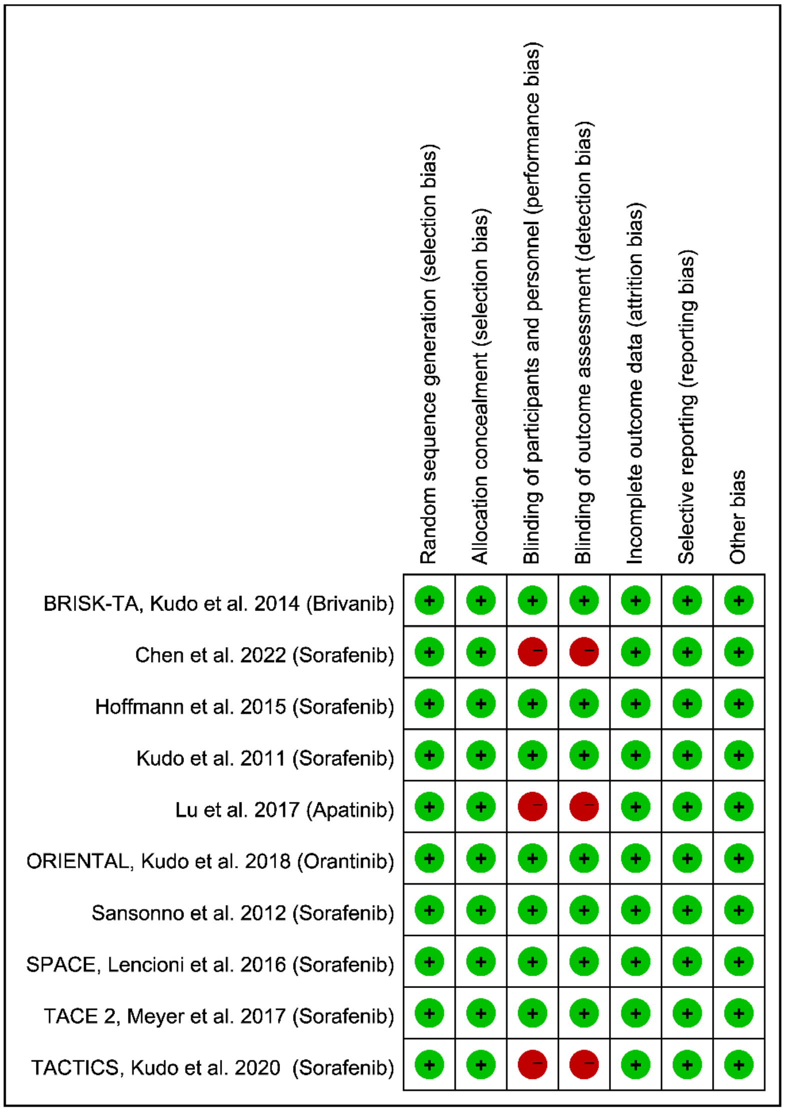

Supplementary Figure 1. Risk of bias assessment of included randomised controlled trials (RCTs) using the Cochrane risk of bias tool.

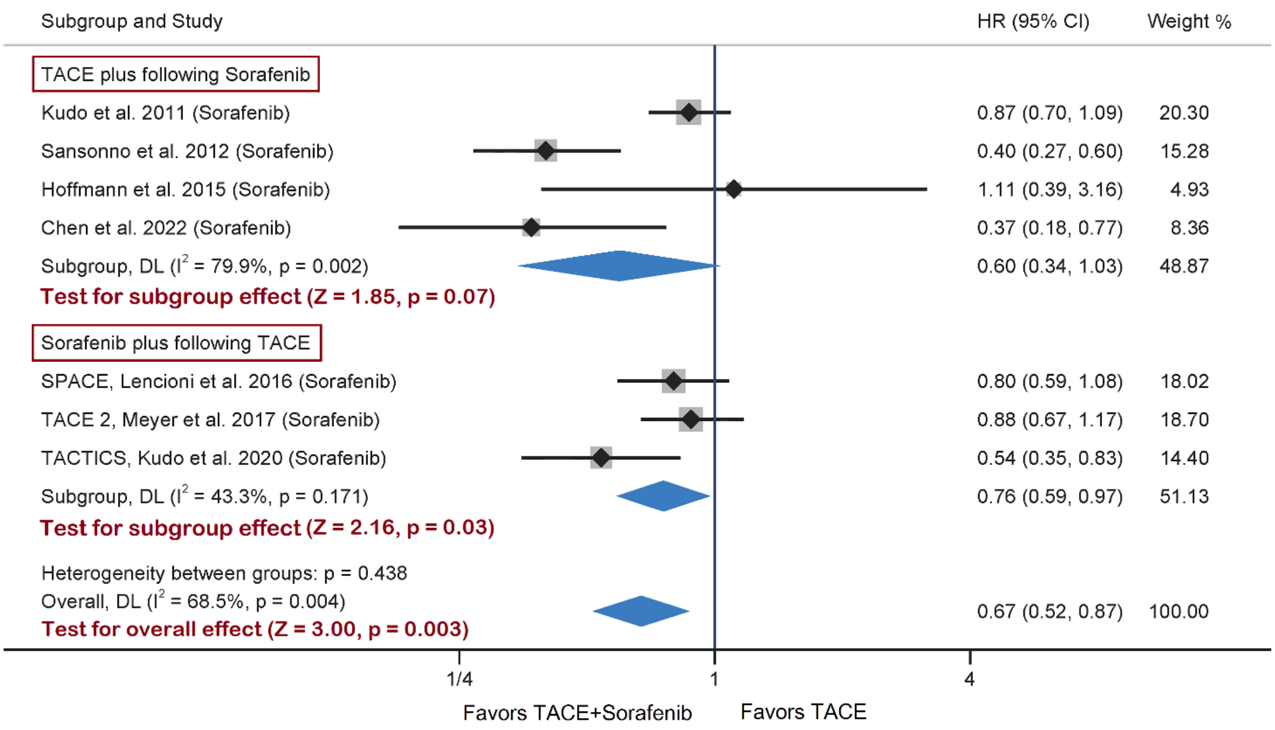

Supplementary Figure 2. Meta-analysis of treatment effects of sorafenib in combination with TACE on TTP in patients with unresectable HCC. For subgroups analysis, 7 trials are divided into two classes based on TACE schedule: TACE plus following sorafenib & sorafenib plus following TACE. The pooled HR of TTP was calculated by using a random-effects model. TACE, transarterial chemoembolization; TTP, time to progression; HR, hazard ratio.

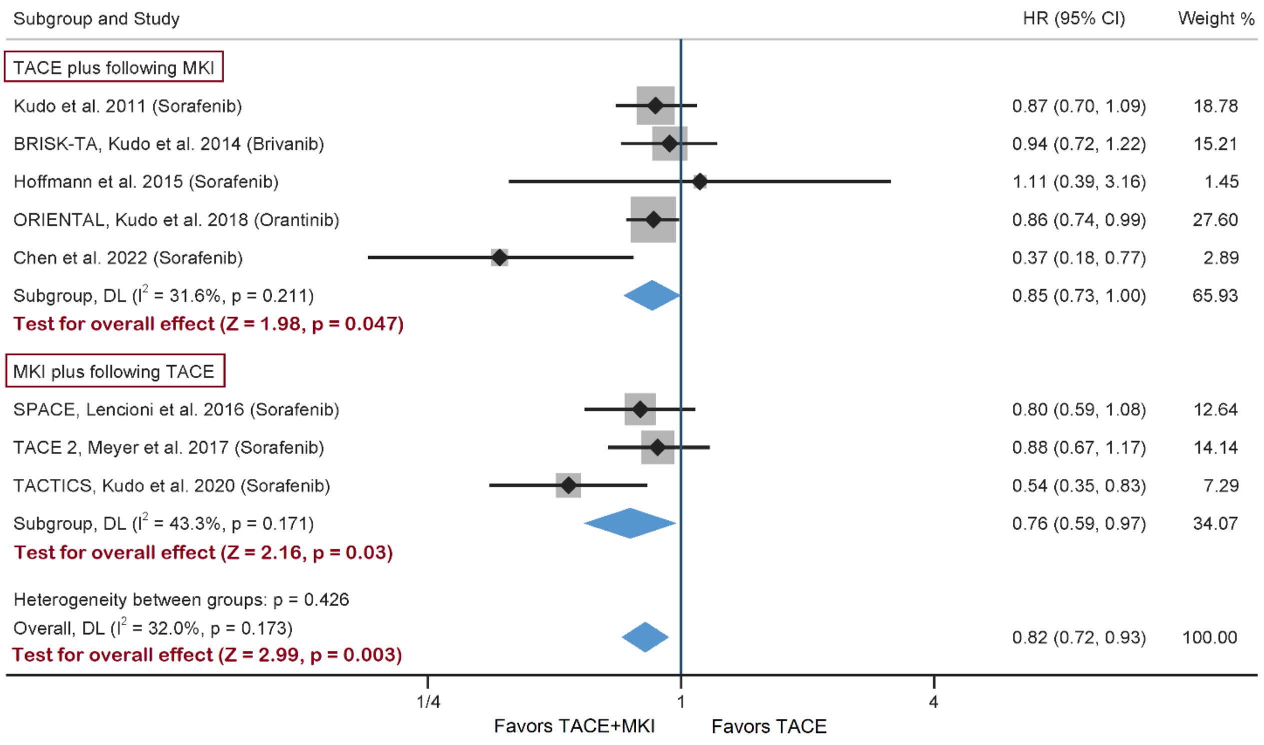

Supplementary Figure 3. Sensitivity analysis for testing TTP was conducted by removing the study of Sansonno et al. which was the source of heterogeneity. The pooled HR of TTP was calculated by using a random-effects model. TACE, transarterial chemoembolization; MKI, multikinase inhibitor; TTP, time to progression; HR, hazard ratio.

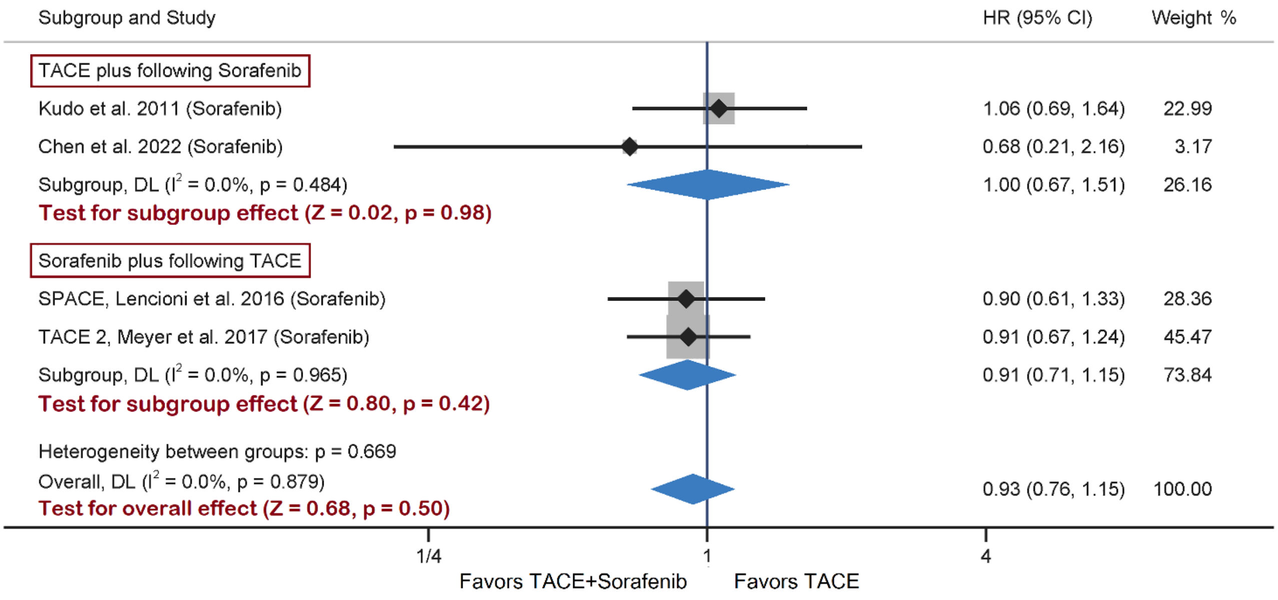

Supplementary Figure 4. Meta-analysis of treatment effects of sorafenib in combination with TACE on OS in patients with unresectable HCC. For subgroups analysis, 4 trials are divided into two classes based on TACE schedule: TACE plus following sorafenib & sorafenib plus following TACE. The pooled HR of OS was calculated by using a random-effects model. TACE, transarterial chemoembolization; OS, overall survival; HR, hazard ratio.

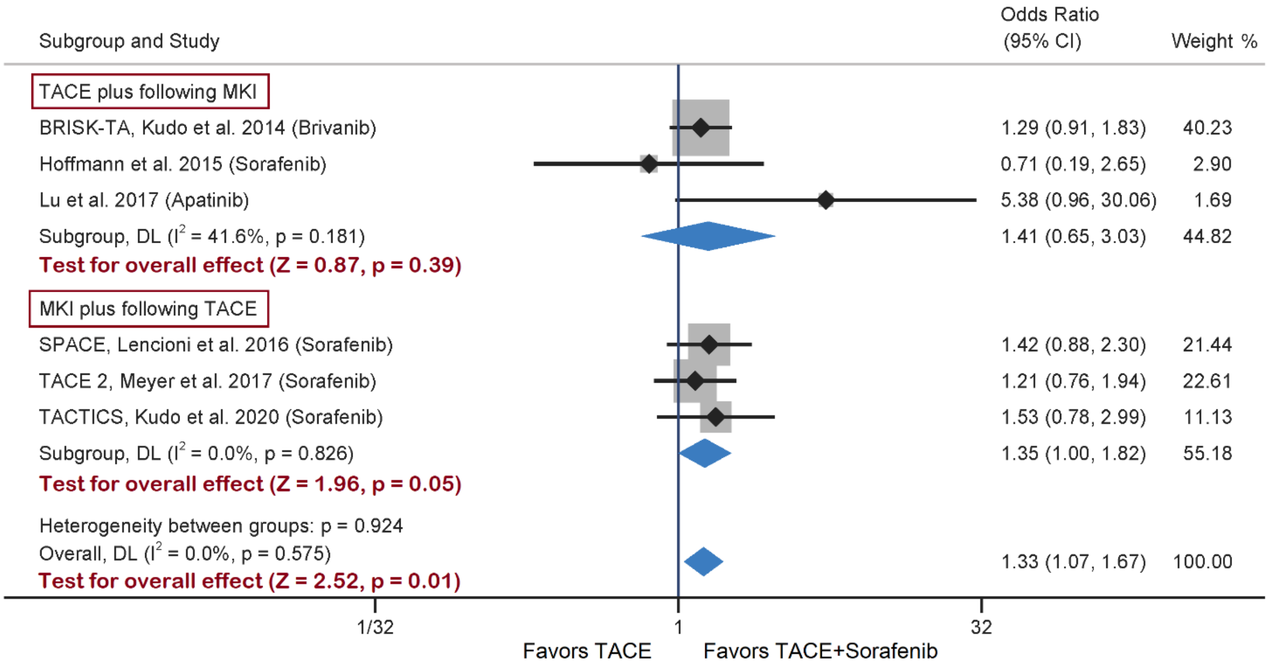

Supplementary Figure 5. Sensitivity analysis for testing ORR was conducted by using odds ratio as summary statistic. The pooled odds ratio of ORR was calculated by using a random-effects model. TACE, transarterial chemoembolization; MKI, multikinase inhibitor; ORR, objective response rate.

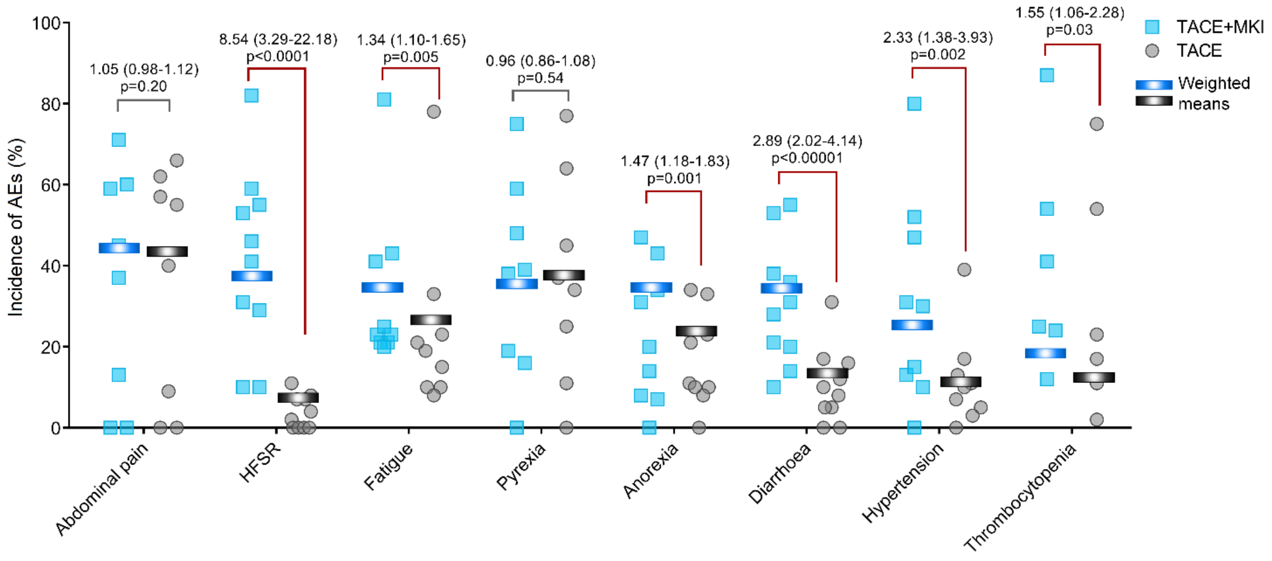

Supplementary Figure 6. Distribution and weighted means of incidence of each AE across trials. The blue boxes and gray dots represented the incidence of each AE from different trials, while the bars represented the weighted means of AE incidence. The risk ratio with 95% CI and p value for each AE obtained from meta-analysis was labeled at the top of symbols. AE, adverse event; HFSR, hand-foot skin reaction; MKI, multikinase inhibitor; TACE, transarterial chemoembolization.

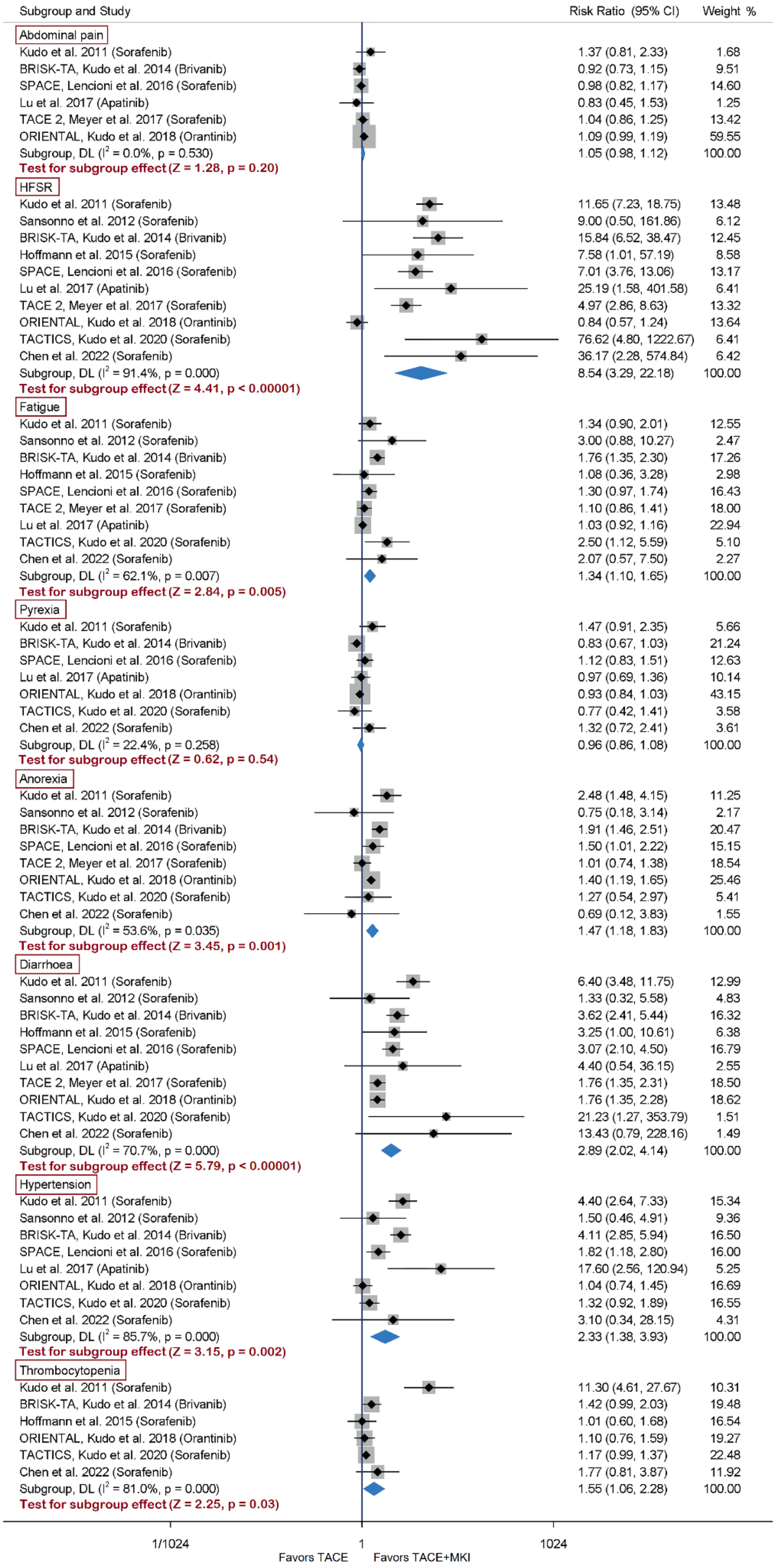
Subgroup and Study
Risk Ratio (95% CI)
Weight %
Abdominal pain
Kudo et al. 2011 (Sorafenib) 1.37 (0.81, 2.33) 1.68
BRISK-TA, Kudo et al. 2014 (Brivanib) 0.92 (0.73, 1.15) 9.51
SPACE, Lencioni et al. 2016 (Sorafenib) 0.98 (0.82, 1.17) 14.60
Lu et al. 2017 (Apatinib) 0.83 (0.45, 1.53) 1.25
TACE 2, Meyer et al. 2017 (Sorafenib) 1.04 (0.86, 1.25) 13.42
ORIENTAL, Kudo et al. 2018 (Orantinib) 1.09 (0.99, 1.19) 59.55
Subgroup, DL (I^2 = 0.0%, p = 0.530) 1.05 (0.98, 1.12) 100.00
Test for subgroup effect (Z = 1.28, p = 0.20)
HFSR
Kudo et al. 2011 (Sorafenib) 11.65 (7.23, 18.75) 13.48
Sansonno et al. 2012 (Sorafenib) 9.00 (0.50, 161.86) 6.12
BRISK-TA, Kudo et al. 2014 (Brivanib) 15.84 (6.52, 38.47) 12.45
Hoffmann et al. 2015 (Sorafenib) 7.58 (1.01, 57.19) 8.58
SPACE, Lencioni et al. 2016 (Sorafenib) 7.01 (3.76, 13.06) 13.17
Lu et al. 2017 (Apatinib) 25.19 (1.58, 401.58) 6.41
TACE 2, Meyer et al. 2017 (Sorafenib) 4.97 (2.86, 8.63) 13.32
ORIENTAL, Kudo et al. 2018 (Orantinib) 0.84 (0.57, 1.24) 13.64
TACTICS, Kudo et al. 2020 (Sorafenib) 76.62 (4.80, 1222.67) 6.41
Chen et al. 2022 (Sorafenib) 36.17 (2.28, 574.84) 6.42
Subgroup, DL (I^2 = 91.4%, p = 0.000) 8.54 (3.29, 22.18) 100.00
Test for subgroup effect (Z = 4.41, p < 0.00001)
Fatigue
Kudo et al. 2011 (Sorafenib) 1.34 (0.90, 2.01) 12.55
Sansonno et al. 2012 (Sorafenib) 3.00 (0.88, 10.27) 2.47
BRISK-TA, Kudo et al. 2014 (Brivanib) 1.76 (1.35, 2.30) 17.26
Hoffmann et al. 2015 (Sorafenib) 1.08 (0.36, 3.28) 2.98
SPACE, Lencioni et al. 2016 (Sorafenib) 1.30 (0.97, 1.74) 16.43
TACE 2, Meyer et al. 2017 (Sorafenib) 1.10 (0.86, 1.41) 18.00
Lu et al. 2017 (Apatinib) 1.03 (0.92, 1.16) 22.94
TACTICS, Kudo et al. 2020 (Sorafenib) 2.50 (1.12, 5.59) 5.10
Chen et al. 2022 (Sorafenib) 2.07 (0.57, 7.50) 2.27
Subgroup, DL (I^2 = 62.1%, p = 0.007) 1.34 (1.10, 1.65) 100.00
Test for subgroup effect (Z = 2.84, p = 0.005)
Pyrexia
Kudo et al. 2011 (Sorafenib) 1.47 (0.91, 2.35) 5.66
BRISK-TA, Kudo et al. 2014 (Brivanib) 0.83 (0.67, 1.03) 21.24
SPACE, Lencioni et al. 2016 (Sorafenib) 1.12 (0.83, 1.51) 12.63
Lu et al. 2017 (Apatinib) 0.97 (0.69, 1.36) 10.14
ORIENTAL, Kudo et al. 2018 (Orantinib) 0.93 (0.84, 1.03) 43.15
TACTICS, Kudo et al. 2020 (Sorafenib) 0.77 (0.42, 1.41) 3.58
Chen et al. 2022 (Sorafenib) 1.32 (0.72, 2.41) 3.61
Subgroup, DL (I^2 = 22.4%, p = 0.258) 0.96 (0.86, 1.08) 100.00
Test for subgroup effect (Z = 0.62, p = 0.54)
Anorexia
Kudo et al. 2011 (Sorafenib) 2.48 (1.48, 4.15) 11.25
Sansonno et al. 2012 (Sorafenib) 0.75 (0.18, 3.14) 2.17
BRISK-TA, Kudo et al. 2014 (Brivanib) 1.91 (1.46, 2.51) 20.47
SPACE, Lencioni et al. 2016 (Sorafenib) 1.50 (1.01, 2.22) 15.15
TACE 2, Meyer et al. 2017 (Sorafenib) 1.01 (0.74, 1.38) 18.54
ORIENTAL, Kudo et al. 2018 (Orantinib) 1.40 (1.19, 1.65) 25.46
TACTICS, Kudo et al. 2020 (Sorafenib) 1.27 (0.54, 2.97) 5.41
Chen et al. 2022 (Sorafenib) 0.69 (0.12, 3.83) 1.55
Subgroup, DL (I^2 = 53.6%, p = 0.035) 1.47 (1.18, 1.83) 100.00
Test for subgroup effect (Z = 3.45, p = 0.001)
Diarrhoea
Kudo et al. 2011 (Sorafenib) 6.40 (3.48, 11.75) 12.99
Sansonno et al. 2012 (Sorafenib) 1.33 (0.32, 5.58) 4.83
BRISK-TA, Kudo et al. 2014 (Brivanib) 3.62 (2.41, 5.44) 16.32
Hoffmann et al. 2015 (Sorafenib) 3.25 (1.00, 10.61) 6.38
SPACE, Lencioni et al. 2016 (Sorafenib) 3.07 (2.10, 4.50) 16.79
Lu et al. 2017 (Apatinib) 4.40 (0.54, 36.15) 2.55
TACE 2, Meyer et al. 2017 (Sorafenib) 1.76 (1.35, 2.31) 18.50
ORIENTAL, Kudo et al. 2018 (Orantinib) 1.76 (1.35, 2.28) 18.62
TACTICS, Kudo et al. 2020 (Sorafenib) 21.23 (1.27, 353.79) 1.51
Chen et al. 2022 (Sorafenib) 13.43 (0.79, 228.16) 1.49
Subgroup, DL (I^2 = 70.7%, p = 0.000) 2.89 (2.02, 4.14) 100.00
Test for subgroup effect (Z = 5.79, p < 0.00001)
Hypertension
Kudo et al. 2011 (Sorafenib) 4.40 (2.64, 7.33) 15.34
Sansonno et al. 2012 (Sorafenib) 1.50 (0.46, 4.91) 9.36
BRISK-TA, Kudo et al. 2014 (Brivanib) 4.11 (2.85, 5.94) 16.50
SPACE, Lencioni et al. 2016 (Sorafenib) 1.82 (1.18, 2.80) 16.00
Lu et al. 2017 (Apatinib) 17.60 (2.56, 120.94) 5.25
ORIENTAL, Kudo et al. 2018 (Orantinib) 1.04 (0.74, 1.45) 16.69
TACTICS, Kudo et al. 2020 (Sorafenib) 1.32 (0.92, 1.89) 16.55
Chen et al. 2022 (Sorafenib) 3.10 (0.34, 28.15) 4.31
Subgroup, DL (I^2 = 85.7%, p = 0.000) 2.33 (1.38, 3.93) 100.00
Test for subgroup effect (Z = 3.15, p = 0.002)
Thrombocytopenia
Kudo et al. 2011 (Sorafenib) 11.30 (4.61, 27.67) 10.31
BRISK-TA, Kudo et al. 2014 (Brivanib) 1.42 (0.99, 2.03) 19.48
Hoffmann et al. 2015 (Sorafenib) 1.01 (0.60, 1.68) 16.54
ORIENTAL, Kudo et al. 2018 (Orantinib) 1.10 (0.76, 1.59) 19.27
TACTICS, Kudo et al. 2020 (Sorafenib) 1.17 (0.99, 1.37) 22.48
Chen et al. 2022 (Sorafenib) 1.77 (0.81, 3.87) 11.92
Subgroup, DL (I^2 = 81.0%, p = 0.000) 1.55 (1.06, 2.28) 100.00
Test for subgroup effect (Z = 2.25, p = 0.03)
1/1024
1
1024
Favors TACE
Favors TACE+MKI

Supplementary Figure 7. Meta-analysis of different kinds of AEs of MKI in combination with TACE in patients with unresectable HCC. The MKI evaluated in these trials included sorafenib, brivanib and apatinib. The pooled risk ratio was calculated by using a random-effects model. AE, adverse event; MKI, multikinase inhibitor; TACE, transarterial chemoembolization.
